# Supplementary material for: Content-rich biological network constructed by mining PubMed abstracts
Source: BMC Bioinformatics. 2004 Oct 8;5:147. doi: 10.1186/1471-2105-5-147 (PMC528731; doi:10.1186/1471-2105-5-147)
Supplement: Additional File 2 — The original results of the above study (non-essential files are deleted to keep the file size under the limit set by BMC bioinformatics). [file 1471-2105-5-147-S2.bz2 › chilibotAdditionalFile2/dip05/591ID9253415E10963/html/TUFA_TSF.html]

 


 **TUFA** and **TSF** 
  
Found 146 abstracts in PubMed, retrieved 05.  
 

 What does Google say? 
 PDF only 
| .edu only 

---

**Interactive relationship** (e.g. stimulation, inhibition, etc)

**Neutral relationship**- Characterization of the interaction between the nucleotide exchange factor EF Ts  [ **TSF** ]  from nematode mitochondria and elongation factor Tu  [ **TUFA** ] .  Ref: 12490713 Nucleic Acids Res, 2002
- The EF Ts  [ **TSF** ]  bound to two eubacterial EF Tu  [ **TUFA** ]  species E.coli and Thermus thermophilus.  Ref: 12490713 Nucleic Acids Res, 2002
- This indicates that nematode EF Ts  [ **TSF** ]  can be used to stabilize homologous EF Tu  [ **TUFA** ]  molecules for experimental purposes.  Ref: 12490713 Nucleic Acids Res, 2002
- In contrast to earlier experiments, purification of free mitochondrial EF Ts  [ **TSF** ]  was accomplished under nondenaturing conditions since only about 60% of the expressed EF Ts  [ **TSF** ]  copurified with E. coli EF Tu  [ **TUFA** ] .  Ref: 12445479 Biochim Biophys Acta, 2002
- Thus, the nematode EF Ts  [ **TSF** ]  appears to have a broad specificity for EF Tu  [ **TUFA** ]  molecules from different species.  Ref: 12490713 Nucleic Acids Res, 2002
- Recombinant nematode EF Ts  [ **TSF** ]  purified from Escherichia coli bound both of these molecules and also stimulated the translational activity of EF Tu  [ **TUFA** ] , indicating that the nematode EF Ts  [ **TSF** ]  homolog is a functional EF Ts  [ **TSF** ]  protein of mitochondria.  Ref: 12490713 Nucleic Acids Res, 2002

**Non-interactive relationship** (e.g. studied together, co-existance, homology, etc.)

- Previous studies have shown that when bovine mitochondrial elongation factor Ts  [ **TSF** ]  EF Ts  [ **TSF** ]  is expressed in Escherichia coli, it forms a tightly associated complex with E. coli elongation factor Tu  [ **TUFA** ]  EF Tu  [ **TUFA** ] .  Ref: 12445479 Biochim Biophys Acta, 2002
- The crystals of the homologous mitochondrial EF Tu  [ **TUFA** ]  EF Ts  [ **TSF** ]  complex diffract to 4 A and belong to space group C2 with cell parameters a=157.7 A, b=151.9 A, c=156.9 A, and beta=108.96 degrees.  Ref: 12445479 Biochim Biophys Acta, 2002
- Isolation, crystallisation, and preliminary X ray analysis of the bovine mitochondrial EF Tu  [ **TUFA** ]  GDP and EF Tu  [ **TUFA** ]  EF Ts  [ **TSF** ]  complexes.  Ref: 12445479 Biochim Biophys Acta, 2002
- Several proteins, including the elongation factors EF G,  **TufA**  and EF Ts  [ **TSF** ] , were found to be highly carbonylated.  Ref: 12845527 Mol Genet Genomics< MedlineTA>Mol Genet Genomics, 2002
- The bovine mitochondrial EF Tu  [ **TUFA** ]  GDP complex, the homologous mitochondrial EF Tu  [ **TUFA** ]  EF Ts  [ **TSF** ]  complex, and the heterologous E. coli mitochondrial EF Tu  [ **TUFA** ]  EF Ts  [ **TSF** ]  complex were isolated and crystallised.  Ref: 12445479 Biochim Biophys Acta, 2002
- Although the EF Ts  [ **TSF** ]  did not bind to bovine mt EF Tu  [ **TUFA** ] , it could bind to a chimeric nematode bovine EF Tu  [ **TUFA** ]  molecule containing domains 1 and 2 from bovine mt EF Tu  [ **TUFA** ] .  Ref: 12490713 Nucleic Acids Res, 2002
- Although the nematode mitochondrial mt EF Tu  [ **TUFA** ]  molecules are extremely unstable and easily form aggregates, native gel electrophoresis and gel filtration analysis revealed that EF Tu  [ **TUFA** ] .EF Ts  [ **TSF** ]  complexes are significantly more soluble.  Ref: 12490713 Nucleic Acids Res, 2002
